# Complete mitochondrial genome of a leaf-mining beetle, *Agonita chinensis* Weise (Coleoptera: Chrysomelidae)

**DOI:** 10.1080/23802359.2017.1365650

**Published:** 2017-08-17

**Authors:** Qingyun Guo, Jiasheng Xu, Chengqing Liao, Xiaohua Dai, Xilin Jiang

**Affiliations:** a Leafminer Group, School of Life and Environmental Sciences, Gannan Normal University, Ganzhou, China;; b National Navel-Orange Engineering Research Center, Ganzhou, China

**Keywords:** Leaf-mining beetle, mitochondrial genome, *Agonita chinensis*, phylogenetic analysis

## Abstract

The complete circular mitochondrial genome of *Agonita chinensis* was 16,395 bp in length, which contained two ribosomal RNA genes, 22 transfer RNAs, 13 protein-coding genes (PCGs) and one non-coding AT-rich region with the length of 2001 bp. All of the 22 tRNA genes displayed a typical clover-leaf structure, with the exception of tRNA^Ser^ (TCT). Twelve PCGs were initiated by ATN codons, except that nad1 started with TTG. Only four PCGs used the typical stop codon ‘TAA’ and ‘TGA’, while nine PCGs terminated with incomplete stop codons (TA or T). Phylogenetic analysis based on 13 PCGs of Chrysomelidae mitogenomes showed that *A. chinensis* was closely related to *Cassida viridis*.

The leaf-mining genus *Agonita* Weise belongs to the tribe Gonophorini (Chrysomelidae: Cassidinae), with 110 species occurring mostly in Oriental region and Ethiopic region (Chen et al. 1986; Staines 2012). *Agonita chinensis* Weise was a newly recorded species in Jiangxi Province, with one generation per year; their larvae fed on the leaves of bamboos such as *Indosasa crassiflora*, and forming a white blotch mine, which could affect leaf photosynthesis and plant growth. However, no mitogenome had been studied for the whole genus of *Agonita*, limited to clarify its phylogenetic relationship with other leaf beetles and sustainable control of the pest. Here, we presented the complete mitochondrial genome of *A. chinensis* (GenBank: MF351622) based on the Illumina pair-end sequencing data.

The specimen was collected in November 2016 at Xiangshan Township, Xunwu County, Jiangxi Province, China (geographic coordinate: N 24°57′55.5″, E 115°45′30.6″). The adult specimen was preserved in 100% ethanol at –80 °C. The total genomic DNA was isolated from head tissue of the adults of *A. chinensis* using Sangon animal DNA extract kit (Sangon Inc., Shanghai, China) following the manufacturer’s instructions. DNA was stored at –20 °C. Raw sequences were assembled into contigs using the Staden Package v1.7.0 (Staden et al. 2000). Two rRNA and all protein-coding genes (PCGs) were annotated by alignment with homologous genes from other published mitochondrial sequences using MEGA 5.0 (Tamura et al. 2011). The transfer RNA (tRNA) genes were predicted using tRNAscan-SE (Lowe and Eddy 1997).

The circular mitogenome of *A. chinensis* was 16,395 bp, AT rich (78.7%), and includes 37 genes for two ribosomal RNA, 22 tRNAs, 13 PCGs and a 2001 bp long non-coding A + T-rich region. Eleven PCGs started with ATN, except that nad1 initiated with TTG. Only four PCGs used the typical stop codon ‘TAA’ and ‘TAG’, while nine PCGs terminated with incomplete stop codons (TA or T), which was commonly reported in other invertebrates (Masta and Boore 2004). All of the 22 tRNAs have a typical clover-leaf secondary structure, except for trnS1 (tRNA^Ser^ (TCT)) lacking a stable dihydrouridine (DHU) stem, which has been observed in several insects’ mtDNA (Kim et al. 2012; Song et al. 2017). The 16S rRNA was 1237 bp long with an AT content of 81.65%, while the 12S rRNA was 723 bp long with an AT content of 81.74%. The non-coding region with an A + T content of 75.71%, was well known for replication initiation (Nardi et al. 2001).

The total length of the 13 PCGs is 11,057 bp, and the overall A + T content of *A. chinensis* PCGs is 78.45%. The concatenated datasets of the 13 PCGs from mitogenome of Chrysomelidae available on GenBank were used to construct phylogenetic relationships by using maximum likelihood methods. Phylogenetic analysis suggested that *A. chinensis* was clustered into the clade including *Rhadinosa nigrocyanea* (Guo et al. 2017), *Cassida viridis*, and *Laccoptera ruginosa* (Figure 1). It had a closer phylogenetic relationship with *Cassida viridis*, which was consistent with morphological classification (Chen et al. 1986; Staines 2012).

**Figure 1.**
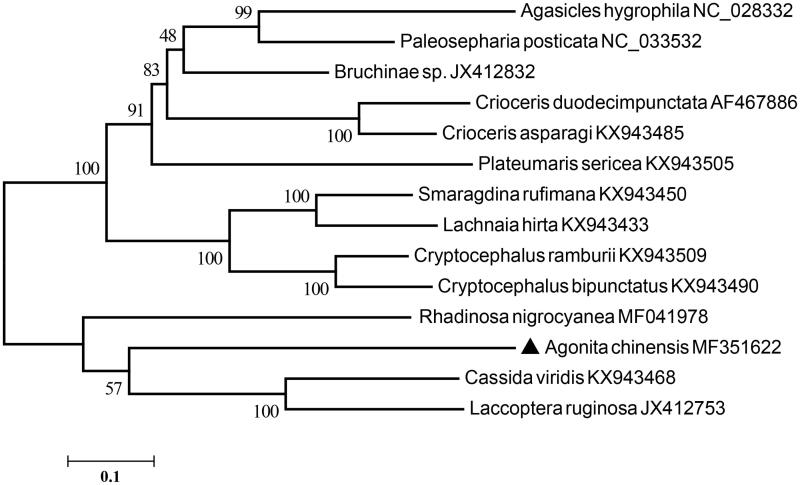
Maximum-likelihood tree of evolutionary relationships *A. chinensis* and 13 other Chrysomelidae species based on mitochondrial PCGs catenated dataset.
